# Physicians and nurses experiences of providing care to patients within a mobile care unit – a qualitative interview study

**DOI:** 10.1186/s12913-024-11517-8

**Published:** 2024-09-13

**Authors:** Christofer Teske, Ghassan Mourad, Micha Milovanovic

**Affiliations:** 1 Department of Health, Medicine, and Caring Sciences, Linköping University, Linköping, Sweden; 2 Department of Emergency medicine in Norrköping, Department of Biomedical and Clinical Sciences, Linköping University, Linköping, Sweden; 3 Department of Internal Medicine in Norrköping, Department of Health, Medicine and Caring Sciences, Linköping University, Linköping, Sweden

**Keywords:** Mobile care, Mobile unit, Nurses, Physicians, Qualitative interviews

## Abstract

**Introduction:**

There is a growing need for alternative forms of care to address citizen demands and ensure a competent healthcare workforce across municipalities and regions. One of these forms of care is the use of mobile care units. The aim of the current study was to describe physicians and nurses experiences of providing care to patients within a mobile care unit in Sweden.

**Method:**

Data were collected between March 2022 and January 2023 through qualitative interviews with 14 physicians and nurses employed in various mobile care units in different regions in Sweden. These interviews were transcribed verbatim and subjected to content analysis, with the study adhering to the Standards for Reporting Qualitative Research (SRQR).

**Results:**

The analysis resulted in two main categories: “Unlocking the potential of mobile care”, and “The challenges of moving hospitals to patients’ homes”; and seven subcategories. The respondents viewed mobile care at home as highly advantageous, positively impacting both patients and caregivers. They believed their contributions enhanced patients’ well-being, fostering a welcoming atmosphere. They also noted receiving more quality time for each patient, enabling thorough assessments, and promoting a person-centered approach, which resulted in more gratifying mutual relationships. However, they experienced that mobile care also had challenges such as geographical limitations, limited opening hours and logistical complexity, which can lead to less equitable and efficient care.

**Conclusions:**

Physicians and nurses in mobile care units emphasized positive outcomes, contributing to patient well-being through a person-centered approach. They highlighted increased quality time, comprehensive assessments, and overall satisfaction, praising the mobile care unit’s unique continuity for enhancing safety and fostering meaningful relationships in the patient’s home environment. In order for mobile care to develop and become a natural part of healthcare, challenges such as geographical limitations and logistics need to be addressed.

**Supplementary Information:**

The online version contains supplementary material available at 10.1186/s12913-024-11517-8.

## Background

Shifts in population demographics and the present structure of the healthcare system prompt inquiries about the optimal care for frail older people [1–4]. The multifaceted health conditions and diverse requirements of these individuals result in increased healthcare appointments and recurrent hospital stays, putting pressure on the current health infrastructure [5, 6]. In Sweden, the state oversees general healthcare policy, with the Inspectorate for Health and Care supervising. While regions ensure that all citizens have access to quality care, municipalities look after long-term health and social care for the frail older people,. Primary care serves as the initial point of contact in the healthcare system, providing basic services either at facilities or at homes. They also guide patients to the appropriate level of care as required [7].

The transition towards accessible and qualitative healthcare is underway in municipalities and regions [8]. This transition is important because some individuals may experience problems accessing healthcare due to of distance, severe illness and immobility [9–11]. This change, however, demands long-term commitment and perseverance, not only from the regions and municipalities, but also from the government [12]. The goal is to develop person-centered, efficient, and purposeful methods that cater to patient needs. This also means that different healthcare stakeholders, specialties, and professions need to collaborate more effectively [6]. To respond to citizens’ demands for accessible care, there’s a need for alternative forms of care, for example, mobile care, that can offer prompt and appropriate care within the available resources [12].

The terminology, i.e. the meaning of mobile care varies from country to country, but the care provided is the same, as is its purpose, to provide highly specialized care, mainly by physicians and nurses, for conditions that normally require hospital admission [13, 14]. Examples of mobile care are geriatric “Hospital at home” programs that offer treatments typically exclusive to hospitals right in the patient’s homes, including monitoring, drug administration, nursing, and rehabilitation processes [13, 15]. Hospital at home is defined as “a service that provides active treatment, by health care professionals, in the patient’s home for a condition that otherwise would require acute hospital in-patient care, always for a limited time period” unlike home nursing care [13, 16]. Patients are evaluated in various settings, including by their general practitioners or in emergency rooms, before being directed to these services. This model can also support those discharged early from hospital [17, 18]. The target group for mobile care varies, but the mobile units in the current study focus on the frail older people. The National Board of Health and Welfare defines frail frail older people as people over 65 years of age with several chronic diseases and extensive needs for both outpatient and inpatient medical care [17].

Transitioning the care of patients from hospitals to their homes poses a formidable challenge, primarily due to concerns regarding patient safety and the constraints inherent to a patient’s home environment. Previous studies show that many patients are sent to hospitals instead of being assessed for mobile care due to various circumstances, e.g., for reasons of convenience [14, 19]. In cases where the assessment is performed, the mobile care team often rejects the patient due to lack of time, logistical reasons or that the patient is unsuitable [13, 14, 20]. More knowledge is needed about physicians and nurses experiences of mobile care to provide an improved and developed perspective on how it can be incorporated into the healthcare system. The aim of the current study was to describe physicians and nurses experiences of providing care to patients within a mobile care unit in Sweden.

## Method

### Design

We employed a qualitative, inductive approach and used a content analysis methodology as outlined by Hsieh & Shannon [21]. In this approach, coding and theme development were driven by the shared meaning found within the data. The design’s primary objective was to discern, analyze, and interpret patterns within the qualitative data. The study adhered to the Standards for Reporting Qualitative Research (SRQR) [22]. The study was accepted by the Ethics Review Authority, Uppsala, Sweden (reg. number: 2020–06986).

### Sampling and setting

The interviews were conducted between March 2022 and January 2023 in four Swedish cities in four different regions with populations varying between 61,000 and 160,000 inhabitants. All cities were equipped with mobile care units. Five units were found through an internet search, after which contact was made with the region management. Of these, four teams agreed to participate. These units specialize in mobile care as their primary field, delivering direct care to patients and offering indirect support to other physicians and nurses involved in providing such care. Mobile care units primarily offer home-based and inpatient care, with the number of patients receiving home care varying from 5 to 15. To be eligible for inclusion, participants had to meet the following criteria: active employment in a specialized field related to internal medicine or geriatric care, a minimum of 2 years of professional experience in the domain of the mobile care unit, and master the Swedish language. Invitation to participate in the study was issued by either the department head or a senior supervising physician within the healthcare facility. All physicians and nurses working in the included mobile care units who fulfilled the inclusion criteria were invited to participate, and all agreed to participate (Table 1).

### Data collection

Due to the COVID-19 pandemic, interviews were performed using telephone (*n* = 11) and Microsoft Teams© (Microsoft Corporation, California, U.S.A) (*n* = 3). Participants were given the opportunity to propose a suitable time for the interview. The interview began with the participant introducing themselves and describing their experience with mobile care. The semi-structured interview guide was created by the authors with open-ended questions and was followed up by probing questions (See supplementary file). One pilot interview was conducted and did not result in any changes to the interview guide and was therefore included in the analysis. All interviews were performed by the first author (CT). CT is a registered nurse working within the field of emergency care and with previous experience in qualitative interviewing. CT had no prior care relationship with the study participants. Participants were encouraged to engage in open discussion, with occasional probing queries aimed at enhancing clarity, such as requests for further elaboration, explanations, and exploration of the how and why aspects. The interviews lasted between 25 and 55 min, were audio-recorded and then transcribed verbatim by CT. Before the study commenced, physicians and nurses were briefed on the study through both verbal and written communication. The participants were assured of confidentiality, and solely the researchers associated with the project could access the data, in line with The Swedish Research Council’s protocols [23].

### Data analysis

The analysis of the transcribed interviews was conducted according to conventional content analysis based on Hsieh & Shannon [21]. All authors individually read four transcripts to gain both depth and breadth in understanding the material. Then, units of meaning in the text that were perceived to capture key thoughts or concepts were marked directly in the text. After this, notes were made in the margins describing the first impression, thereby conducting an initial analysis. To increase the trustworthiness of the study, all authors individually coded four transcripts and then mutually discussed the findings to employ a consistent coding scheme. Based on this coding scheme, CT coded the rest of the transcripts. The codes were then sorted into subcategories based on how the different codes were related and linked to each other. These subcategories were thereafter used to organize and group codes into meaningful clusters, which formed the basis for the emerging subcategories. Depending on how the subcategories were related to each other, they were afterwards divided into a smaller number of categories. These steps were mutually discussed by all authors. The findings of the research were strengthened and clarified by using specific quotations. These selected pieces, derived directly from the initial dataset, were eventually translated into English. Table 2 provides examples representing different stages of the analysis.

## Results

The results are derived from interviews with physicians and nurses, who were actively employed in specialized fields related to internal medicine or geriatric care. Each participant had at least two years of professional experience in the mobile care unit and was proficient in the Swedish language. Analysis of the interviews resulted in two main categories and seven subcategories according to Table 3. The main categories were: Unlocking the potential of mobile care and The challenges of moving hospitals to patients’ homes.

### Unlocking the potential of mobile care

Physicians and nurses described that mobile care promotes person-centered care based on mutual equality. Caring for the patient in their home increases transparency and safety for patients. Cooperation with different treatment units ensures comprehensive and safe care. It is a healthy work environment that gives professional pride.

### Person-centered: the right way to care

Physicians and nurses described that it was rewarding to observe the patient in their natural environment. Physicians and nurses who had previously worked in a hospital setting experienced a shift in the balance of power when care had taken place in the patient’s home. The healthcare staff described that they felt that they were not in a position of power and called it “mutual equality”, and that this led to patients being more inclined to open up and share their opinions. This contributed to a more accurate assessment that aligned with a person-centered care approach. In an assessment of the patient in their living environment, physicians and nurses had been able to identify potential obstacles and complications more effectively. Such obstacles might have been, for example, thresholds in the dwelling that could potentially have been a fall risk. A significant distance between the toilet and bedroom might have resulted in the patient avoiding diuretics due to concerns about incontinence. Physicians and nursesdescribed that it is of central importance to not only identify existing shortcomings but also to anticipate potential vulnerabilities that might have arisen during the period when the patient was enrolled in the mobile care unit. Proactively working on prevention had been essential to ensure the patient’s overall well-being.

*“I find it very rewarding to enter their home environment. You sort of get on the same wavelength*,* and it feels*,* what should I say*,* more human to sit with them at home. You get a sense of how this patient operates in their home environment*,* and it’s important information that we lack when the patient is in their hospital room”* [7].

### Safer care through increased patient activity

Physicians and nurses described that patients are satisfied with being cared for at home. The care can be planned collaboratively to a greater extent, ensuring continuous patient involvement. It facilitates conducting examinations and treatments at home rather than needing transportation. Physicians and nurses shared their experiences of safety of care and that a factor for increased safety of care was to enable a care plan with the patients. They expressed that this form of care offers a different type of continuity compared to hospital care where there is variability in the staff. Knowing the patient and their history increased the safety of care. According to physicians and nurses, communication was a key factor. It was essential to inform patients about the reason for the unit’s visit and the necessary treatments Additionally, informing relatives was highlighted as a aspect of care. Physicians and nurses described that relative need to be involved and aware of the plan for the patients, especially since this form of care might be new to some. Furthermore, it was important for physicians and nurses that they provide information to both relatives and the patient on how to contact healthcare if required as this leads to increased security for them.

*“Sometimes*,* they may need an injection to reduce fluid retention for a week*,* and then the nurse will work together with the patient to develop a plan so that they feel confident in saying*,* ‘Yes*,* now we’re going to do it like this”* [10].

### Good care requires good collaboration

To ensure high-quality care, collaboration within different healthcare organizations was essential according to physicians and nurses. They conveyed that frequent interaction between various healthcare entities and professions enhanced the sense of security for physicians and nurses, which in turn positively affected the patients. When the mobile unit was aware that home care services assisted or that home healthcare was responsible for the patient at night, the unit felt an increased sense of security in providing care in the patient’s home.

*“But the idea and the goal are that patients who do not require inpatient care should be able to stay with our assistance and in collaboration with home healthcare*,* as well as with*,* for example*,* occupational therapists and physiotherapists”* [11].

The perceived benefit of collaborating with hospital specialists, who are not directly part of the mobile unit, was perceived to facilitate the unit’s care delivery. A contributing factor to effective collaboration was that the facility was a smaller hospital, and the mobile unit was stationed close to the hospital’s departments.

*“We are a very small hospital*,* so we have the advantage of being close at hand. We have cooperation among all in.”* [9].

### Making a difference gives a sense of professional pride

Physicians and nurses experienced that they were doing something good for the frail older people. They provided good healthcare in a place where the patient wanted to be. Physicians and nurses believed that care in a patient’s home environment surpassed the care that was provided in hospitals. They felt that they had a meaningful profession and that they had a impact on the patients’ lives, but they also perceived that they contributed to the patient’s well-being. Physicians and nurses perceived that they contributed to the patient’s well-being. Physicians and nurses described that they had more time for each patient and did not have to move between patients as they did in the hospital. This led to less stress. It also allowed for a thorough assessment and promoted the establishment of a more rewarding mutual relationship.

*“I believe that it’s necessary for us to fulfill a role and make a contribution for the elderly. I see that the unit is needed and that we serve a purpose”* [3].

### The challenges of moving hospitals to patients’ homes

Physicians and nurses describe that geographical differences and the limited operating hours of mobile care teams lead to unequal care. They face logistical challenges, such as transporting equipment and navigating different administrative systems, which need improvement. Additionally, maintaining good hygiene in less clean home environments can be difficult.

### Mobile care availability varies among different populations

Physicians and nurses emphasized the limitations of a mobile care unit compared to traditional hospital care. They often used expressions such as: “compared to the hospital or the emergency room”.

Some of the physicians and nurses highlighted that this type of care is limited to geographical boundaries. Within a municipality, there is often a higher concentration of resources and opportunities compared to areas outside the central parts of the municipality. Physicians and nurses described that if the patients live within the area of the unit, they will be offered this type of care, otherwise not, leading to inequality in care. Furthermore, mobile care was perceived as insufficient as the number of scheduled visits must be reduced if the travel time becomes too long. At most, physicians and nurses need to travel up to 60 minutes for a visit.

*“There are still quite significant differences in the care one receives when living inside the city as opposed to living outside the municipality.”* [1].

*“The furthest locations. It’s travel time and such. Considering that*,* we are not very efficient.”* [3].

### Limitations due to the unit size and working hours

According to physicians and nurses, the mobile unit usually consists of a fixed number of employees who are not replaced when illness occurs, making the unit fragile. The units’ operations include both scheduled and emergency visits, and emergency visits can be limited due to lack of necessary resources, e.g. due to illness in the unit members. In such situations, the common alternative is to call for ambulance transportation that brings the patient to nearest hospital for an emergency assessment.

Another aspect is that the mobile unit is only available during office hours. If the patient experiences an emergency with their health outside the office hours, they could speak to a healthcare professional who works in a hospital. Physicians and nurses perceived this opportunity as positive, that it provided an extra security for patients connected to mobile care, while others were more negative to the limited opening hours compared to the hospital.

*“We work regular office hours*,* Monday to Friday. Then during other times*,* they can call us*,* and we leave a brochure. And if we don’t answer the phone*,* they are redirected to the department*,* so they can get in touch with the doctor. It has never really become a problem.”* [8].

### The importance of equipment and logistics

Physicians and nurses described that conducting home visits required extensive preparation, especially concerning the equipment that needed to be brought along. Technical complications can arise, which may be difficult to address in the patient’s home, underscoring the importance of reliable equipment. Another challenge highlighted by physicians and nurses was the incompatibility in record-keeping systems across different forms of care. Standardizing these systems could optimize the workflow. Moreover, physicians and nurses emphasized that some medical equipment cannot be easily implemented in the home environment. These were for example monitoring equipment, including the tracking of vital functions, and infusion systems that administer intravenous drugs safely.

*“It requires quite a bit of logistics. You have to bring things with you. I realized it now when I was about to leave. It demands logistics*,* and you have to be organized.”* [9].

Som physicians and nurses made it clear that not all patients are suitable for a specific treatment at home. In situations where the patient’s condition requires intravenous treatment, but the patient lacks supervision or municipal interventions, the unit need to make an assessment. If the unit can be present during the entire treatment period, then it is safe for the patient to receive the treatment at home, otherwise the alternative is to go to hospital.

Another issue was hygiene problems experienced by physicians and nurses. For example, in wound dressings, it is difficult to maintain cleanliness if the home is already dirty, which normally is not a problem in the hospital environment.

*“First*,* it’s about how the home looks and what possibilities there are. If the home is in disarray*,* it’s impossible to keep it clean. I know*,* I was sewing today*,* and when I compare it to the healthcare center*,* it’s quite sterile in comparison to a bedroom”* [2].

## Discussion

To our knowledge, this is the first study describing physicians and nurses’ experiences with providing care to patients within a mobile care unit in Sweden. The study contributes valuable knowledge and insights into how Physicians and nurses experience this type of highly specialized care in the patients’ homes, which differs from home care nursing which mainly offers basic medical treatment such as health monitoring, medication administration, wound dressing, and overall patient health support. Physicians and nurses considered that mobile care in the home environment offers advantages that have a positive impact on both the patient and physicians and nurses themselves. However, they also expressed some challenges connected with mobile care.

Physicians and nurses described mobile care as a person-centered approach, where caring for patients in their own home has several positive aspects that benefit not only the patient but also physicians and nurses. They perceived it as gratifying to witness patients in their natural surroundings and noted a power shift during home care, fostering mutual equality, which they felt was difficult to achieve when they worked in hospitals. Physicians and nurses described that patients experience satisfaction when they receive care at home. They emphasized that mobile care is characterized by collaborative planning, which ensures continuous patient participation. Although person-centered care emphasizes the importance of patient involvement in decision-making [24], earlier research has shown that not all patients prefer active participation. [25, 26]. This is mainly due to health-related limitations, lack of support from physicians and nurses, or unfamiliarity with the possibility of participate actively. However, in cases where patients want to participate actively, they feel opposed by physicians and nurses. In those moments, they might feel like they don’t have much say or control, and it can make them feel less powerful and independent [27, 28]. This suggests that physicians and nurses should pay attention to patients’ needs and wishes for participation in their care. It is also valuable to address non-active participation through targeted efforts such as patient education and empowerment initiatives to facilitate a smooth transition to acceptance of person-centered care in the home environment [26]. Through these efforts, we believe that it is possible to further promote and implement a person-centered approach in mobile care.

Physicians and nurses described that they received more quality time for each patient, enabling a more comprehensive assessment and fostering a more satisfying person-centered care. Specifically, they believed that their contributions had a substantial impact on the patient’s overall well-being and perceived a consistent sense of welcome, receiving affirmative responses regarding their endeavors. Physicians and nurses experience that the mobile care unit provides a unique continuity compared to hospital care, where staff turnover can introduce variability. Getting to know the patient and their medical history contributes to enhanced safety in care delivery. Previous research [11–13] has shown that building and maintaining relationships with the frail older people with physicians and nurses can be challenging due to the specialized and fragmented healthcare system. A limited number of staff meeting patients in their home environment usually means consistent contact that promotes the quality of care, affecting patients’ feelings of safety and comfort. However, other studies show that patients receiving medical care at home tend to report higher levels of satisfaction with their treating physician compared to patients receiving care in a traditional acute hospital environment [12–14]. Physicians and nurses in this study advocate for the mobile care unit, citing its unique continuity compared to hospitals. We therefore assume that consistent contact with a limited number of staff promotes relational continuity, positively impacting patient satisfaction.

Healthcare professional described that they provided good healthcare in a place where the patient wanted to be. They believed that care in a patients’ home environment surpassed the care that was provided in hospitals and had an impact on the patients’ lives. Physicians and nursesalso described having more time for each individual patient. This allowed for a thorough assessment and promoted the establishment of a more rewarding mutual relationship. This suggests that physicians and nurses appreciated the work environment in the mobile care team. Previous studies have shown a positive correlation between a healthy work environment and better patient experiences [29, 30]. This implicates that a positive work environment in mobile care has far-reaching implications that extend beyond just the well-being of physicians and nurses. It also positively influences patient satisfaction, quality of care, staff engagement, and the overall efficiency of healthcare delivery in the mobile setting.

Physicians and nurses also described challenges in the work environment including unsanitary living conditions that can worsen a patient’s medical condition and make infection control more difficult. Previous studies confirm that there is an increased risk associated with certain types of treatment at home and that it is important to make a careful assessment of whether the patient and the environment are suitable for care [14]. On the other hand, being hospitalized, increases the risk ofnosocomial infections [31–33].

Physicians and nurses described that the mobile care units have limitations in terms of accessibility. This mostly concerns geographic accessibility, where patients in rural areas do not have the same opportunity for mobile care as in the cities. They also described that the units’ working hours and travelling distances was a limiting factor. The availability of mobile care, both geographically and in terms of restricted opening hours, is not in line with the Healthcare Act in Sweden [17], which stipulates that healthcare should be provided on equal terms for the entire population. Geographical accessibility can however be challenging to fulfill as Sweden is sparsely populated compared to many other European countries [34]. Proximity to patients in rural areas is a crucial factor affecting access to primary care [19, 20]. To address this issue, the Ministry of Social Affairs has been tasked by the government to investigate and propose changes to increase access to healthcare in rural areas [21]. Global observations indicate a variety of essential approaches for enhancing accessibility to primary healthcare services in rural areas. These encompass reinforcing the healthcare financing system, enhancing the availability of medicines and supplies, collaborating with diverse partners and communities, implementing a robust evaluation system, and fostering dedicated leadership [35–37]. This indicates that follow-up healthcare appointments, digital solutions may become more relevant in the future to minimize transportation for the mobile care units.

### Strengths and limitations

Mobile care is not yet widely adopted as a working method in Sweden. Consequently, a geographic spread could not be achieved, and the number of participants was limited. Nevertheless, the findings in this study are based on data collected from a relatively high number of physicians and nurses in Sweden with experiences of working in different mobile care units. According to Malterud [38], this indicates that the study has achieved sufficient information power, as all physicians and nurses working in the mobile care units participated in this study. They contributed with their unique experience and provided valuable knowledge to answer the aim of the study. Furthermore, the interviews yielded consistent data since no new information appeared in the last interviews, and this data was analyzed using an established analysis strategy by Hsieh & Shannon [21].

Eleven of the interviews were carried out via telephone and three via video using Microsoft Teams©. This might be considered as a limitation as telephone interviews may have impacted the richness of interview content compared with video interviews. However, research shows that the difference between telephone and video interviews is modest [39, 40]. One strength is that the first author (CT) conducted all interviews, which may have influenced the quality of the interviews positively as the interviewer’s interview technique improved with each interview. Another strength is that all authors individually coded four transcripts and mutually discussed the findings to employ a consistent coding scheme, that CT used to code the rest of the transcripts afterwards. Furthermore, all authors participated in forming subcategories and categories to ensure credibility. Dependability was established by maintaining a comprehensive audit trail, ensuring consistent coding procedures, and involving multiple analysts to verify the stability and reliability of the findings, with every step of the research process thoroughly documented in the methods section.Variations were discussed among the authors during the meetings for the data analysis to enhance the confirmability of the study. The authors have different backgrounds and expertise, i.e. nursing, medicine and biomedicine and this can be seen as an “investigator triangulation” and thus a strength [41].

Although other mobile care units may work differently and have other experiences, our findings may demonstrate transferability to this context as care is delivered to patients in their homes, even though this could differ in content and delivery mode.

## Conclusions

Physicians and nurses experience mobile care as a person-centered approach, promoting holistic care and collaborative planning. It emphasizes ongoing patient participation and eliminated transportation needs. On the other hand, mobile care poses challenges such as inequality of care if patients live outside the units’ areas, incompatible record-keeping, and difficulty implementing the use of certain medical devices. Despite this, mobile care is considered a a good alternative to traditional hospital care, where physicians and nurses felt they had a meaningful profession that positively affects the lives and well-being of the patients, and thus fostering rewarding mutual relationships. The challenge for the future is to engage at a national level with physicians, managers, and politicians to achieve improvements. Failing to come together to develop care pathways relevant to rural communities, for example, could be missing an opportunity to improve the nation’s health.

**Table 1:** Demographic data of the participating physicians and nurses

	Physician	Nurse
**Ocupation**	4	10
**Gender Male/Female**	3/1	-/10
**Mean age of experience in a Mobile care unit (range)**	3.25 (2–7)	3.1 (2–7)

**Table 2:** Examples of the analyzing process

Interview excerpts (interview number, side number and line number)	Generating initial codes of relevance to the research question	Defining and naming subcategories	Main Categories
**Interview no 7**: I find it very rewarding to be in their home environment; you somehow get on the same wavelength, and it feels, what should I say, more humane to sit with them at home	Human encounter	Patient-Centered: The Right Way to Care	Unlocking the Potential of Mobile Care
**Interview number 9**: There is a geographical limitation, and it is that there is a population living far out on the islands here in the municipality, and we, well, started with a two-mile limit. But now it’s probably a five-mile limit.	Geographical limitation	Mobile care availability varies among different populations	The challenges of moving hospitals to patients’ homes

**Table 3:** Main categories and subcategories

Category	Subcategories
Unlocking the potential of mobile care	- Patient-Centered: The right way to care - Patients highly active in care - Good care requires good collaboration - Making a difference gives a sense of professional pride?
The challenges of moving hospitals to patients’ homes	- Mobile care is not available to everyone. - Mobile care only cares daytime. - The importance of equipment and logistics

## Electronic supplementary material

Below is the link to the electronic supplementary material.

Supplementary Material 1

## Data Availability

The datasets used and/or analysed during the current study cannot be shared openly but are available on request from authors.
