# Supplementary material for: Physicians and nurses experiences of providing care to patients within a mobile care unit – a qualitative interview study
Source: BMC Health Serv Res. 2024 Sep 13;24:1065. doi: 10.1186/s12913-024-11517-8 (PMC11401412; doi:10.1186/s12913-024-11517-8)
Supplement: Supplementary file 1 — Supplementary Material 1 [file 12913_2024_11517_MOESM1_ESM.docx]

| Interview guide |
| --- |
| 2. **Can you describe the patients who benefit from your services?**  How can the patient connect with your unit?  How do you think patients experience being cared for by a mobile unit?  What does the unit do to ensure that the patient feels secure with the care they receive? |
| 3. **How do you experience caring for patients who are connected to the mobile unit?**  Can you describe a typical day for you?  What tasks do you have?  What type of care can't be provided by your unit and why? |
| 4. **To conduct the care you provide, which organizations do you collaborate with?** How does it work? |
| 5. **What opportunities and limitations do you see in mobile healthcare in the future?** |
